# A Novel and Efficient Five-Component Synthesis of Pyrazole Based Pyrido[2,3-*d*]pyrimidine-diones in Water: A Triply Green Synthesis

**DOI:** 10.3390/molecules21040441

**Published:** 2016-04-01

**Authors:** Majid M. Heravi, Mansoureh Daraie

**Affiliations:** Department of Chemistry, School of Science, Alzahra University, Vanak, Tehran 1993893973, Iran; daraie_m@yahoo.com

**Keywords:** pyrazolo[4′,3′:5,6]pyrido[2,3-*d*]pyrimidine-diones, green synthesis, multi-component reaction, in water reaction

## Abstract

A novel one pot synthesis of pyrazolo[4′,3′:5,6]pyrido[2,3-*d*]pyrimidine-diones, via a five-component reaction, involving, hydrazine hydrate, ethyl acetoacetate, and 1,3-dimethyl barbituric acid, an appropriate aryl aldehydes and ammonium acetate catalyzed via both of heterogeneous and homogeneous catalysis in water, is reported.

## 1. Introduction

Among the known infectious diseases, tuberculosis (TB) [1] and malaria [2] are the most distressing and devastating, both from impermanence and sickness points of view. Malaria and TB are primeval and prolonged infectious diseases initiated chiefly by the parasite *Plasmodium falciparum* and the pathogen Mycobacterium tuberculosis (MTB), respectively [3,4].

Electron-rich nitrogen heterocycles, such as some pyrimidine derivatives, are well established as antimalarial agents [5]. They also play key roles in diverse biological activities. For instance, a wide range of pyrimidine derivatives show other widespread biological activities and are being used as pharmaceuticals for industrial and prescribed drugs [6]. They show potencies such as Tie-2 kinase inhibitors [7], HIV-1 inhibitors [8], adenosine A1 receptor antagonism [9], anticancer agents [10], analgesics [11], cardiovascular agents and anti-allergic agents [12].

Facile and green synthetic approaches are an important issue in organic synthesis. The generation of divergent and complicated molecules from either commercially available or readily accessible starting materials is an inspiring theme in contemporary organic synthesis [13]. The compatible combination of multi-component reactions (MCRs) and unconventional reaction conditions has recently attracted much attention and stirred up the interest of synthetic organic chemists, resulting in concurrent advance and growth of both MCRs and green chemistry toward ideal synthetic chemistry [14,15]. Multi-component reactions (MCRs), in which multiple reactions are joined in a single synthetic operation performed in one pot, have been extensively and broadly employed in the total synthesis of natural products and their building blocks [16]. Using such strategies for the synthesis of a desired target avoids purification of different precursors as well as tedious protection and deprotection of functional groups frequently, required in multistep synthesis. Due to the shortening of the reaction steps and atom economy involved, a higher degree of component-multiplicity leads to greener conditions.

Nowadays, green chemistry has become a main motivation and inspiration for organic chemists to develop eco-friendly and benign pathways for the synthesis of organic compounds, particularly those exhibiting biological activity [17,18]. Manipulation of multi-component reactions, performed in water as a green and abundant solvent, is another attractive concern of organic synthetic chemists, complying with green chemistry principles. A wide variety of divergent chemical transformations can occur in water. Breslow [19], Li [20], Kobayashi [21], Sharpless [22] and many other renowned organic chemists have momentously extended the number of reactions that can be conducted in water. Reaction in water offers unique reactivity and selectivity. Moreover, water can be readily separated and isolated from organic materials by simple procedures.

Another latent opportunity for in water synthesis is the development of a high atom economy that can facilitate catalyst recovery, recycling and simple product isolation. Idyllically, the reactant and the product should have no or very little water-solubility. Thus, the product can be isolated by simple phase separations and the catalyst can be easily recycled. As a matter of fact, in such circumstances, it is possible to recycle the catalyst-bearing aqueous solution for a prolonged period of time without the requirement either discharging it or recreating it, providing the low atom-efficiency process that resulted in the accretion of undesired materials in the solution.

Heterogeneous catalysis is of paramount importance in green chemistry, due to simplicity of separation, recovery and recyclability of most heterogeneous catalysts [23,24].

Pyrazolo based pyrido[2,3-*d*]pyrimidine-diones and their derivatives are a very important class of heterocycles with a wide range of pharmacological and biological activities.

The synthesis of pyrazole based pyrido[2,3-*d*]pyrimidine-diones was reported in 2012 and 2014 via a three component reaction using barbituric acids, 1*H*-pyrazol-5-amines and differently substituted aromatic aldehydes using PTSA (*p*-Toluenesulfonic acid) under solvent-free conditions, IL-cells (cellulose supported ionic liquid) and ethanol have been reported respectively [25,26].

We are interested in heterocyclic chemistry and chemical and molecular diversity in this field [27,28,29,30,31,32,33,34]. In continuation of our investigation on the synthesis of heterocyclic compounds using different heterogeneous nanocatalysts in organic reactions and, in particular, different heterocyclic systems [35,36,37,38,39,40,41,42,43,44], and expansion of our work on multi-component reactions and green chemistry [45,46,47,48,49,50,51,52,53,54], herein, we report an efficient triply green protocol for the synthesis of pyrazole based pyrido[2,3-*d*]pyrimidine-diones.

## 2. Results

**Scheme 1:**
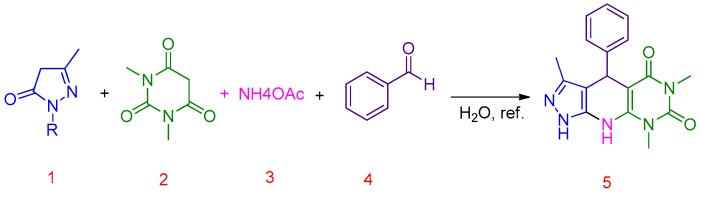
Model reaction for pyrazole based pyrido[2,3-*d*]pyrimidine-dione synthesis.

Initially, we designed a one pot four-component reaction, employing different starting materials leading to pyrazole based pyrido[2,3-*d*]pyrimidine-diones in the absence of any catalyst in water (Scheme 1).

In this strategy, as a model reaction, we used 3-methyl-pyrazolone 1, 1,3-dimethyl barbituric acid 2, ammonium acetate 3 and benzaldehyde 4 in refluxing water. This reaction was monitored by TLC (Thin layer chromatography), showing no progress.

Thus, we examined the reaction in the presence of various catalysts in different solvents under mild reaction conditions (Table 1).

Considering this point that has now been turned into a fact, nowadays, a plethora of nanometal oxides are used as efficient catalysts in several of the organic transformations. Among other catalysts examined, we initially used ZnO and then selected nano-ZnO from analyzing our results obtained from sets of reactions, as a green catalyst. We have also found that the amount of our model reaction, and, in general, 0.04 g of nano-ZnO is the optimum amount of catalyst. Under optimized reaction conditions, using optimal amounts of nano-ZnO as heterogeneous catalyst in boiling water as solvent, we designed this four component reaction, providing the corresponding pyrazolo-[4′,3′:5,6]pyrido[2,3-*d*]pyrimidine-diones in high yield.

As far as green chemistry is concerned, our methodology was superior than that of the preparation of pyrazole based pyrido[2,3-*d*]pyrimidine-diones, using PTSA, previously [25].

When PTSA is heated with acid and water, a hydrolysis reaction takes place and toluene is formed along sulphuric acid.

Our strategy also looks more promising when compared with the recently reported synthesis of pyrazole based pyrido[2,3-*d*]pyrimidine-diones, using pre-prepared IL-Cells [26] as catalyst, due to the commercial availability of nano-ZnO.

**Figure 1:**
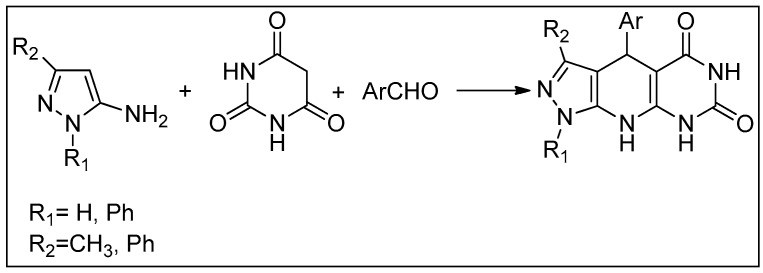
Synthesis of pyrazole based pyrido[2,3-*d*]pyrimidine-diones [25,26].

The reaction pathway for both strategies are the same and depicted in Figure 1.

Under optimized conditions, using nano-ZnO as heterogeneous catalyst in boiling water as solvent, this four component reaction gave the corresponding pyrazolo-[4′,3′:5,6]pyrido[2,3-*d*]pyrimidine-diones in excellent yield (Table 2).

Although the literature survey discloses a plethora of multi-component reactions, five-component reactions are scarcely found [55,56,57,58,59,60,61].

**Scheme 2:**
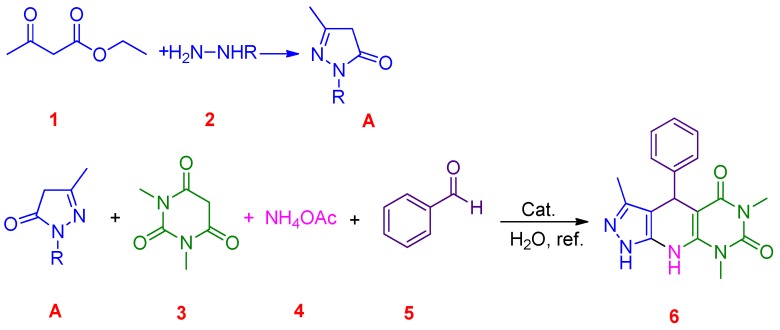
Five-component reaction for pyrazole based pyrido[2,3-*d*]pyrimidine-dione synthesis.

During our practical work, we observed that the reaction of ethyl acetoacetate and hydrazine hydrate proceeded smoothly and was completed rapidly [62,63] to afford 3-methyl-5-hydrazolone, virtually in quantitative yield. Thus, we decided to investigate the synthesis of pyrazolo-[4′,3′:5,6]pyrido[2,3-*d*]pyrimidine-diones via a one pot five component reaction (Scheme 2).

**Scheme 3:**
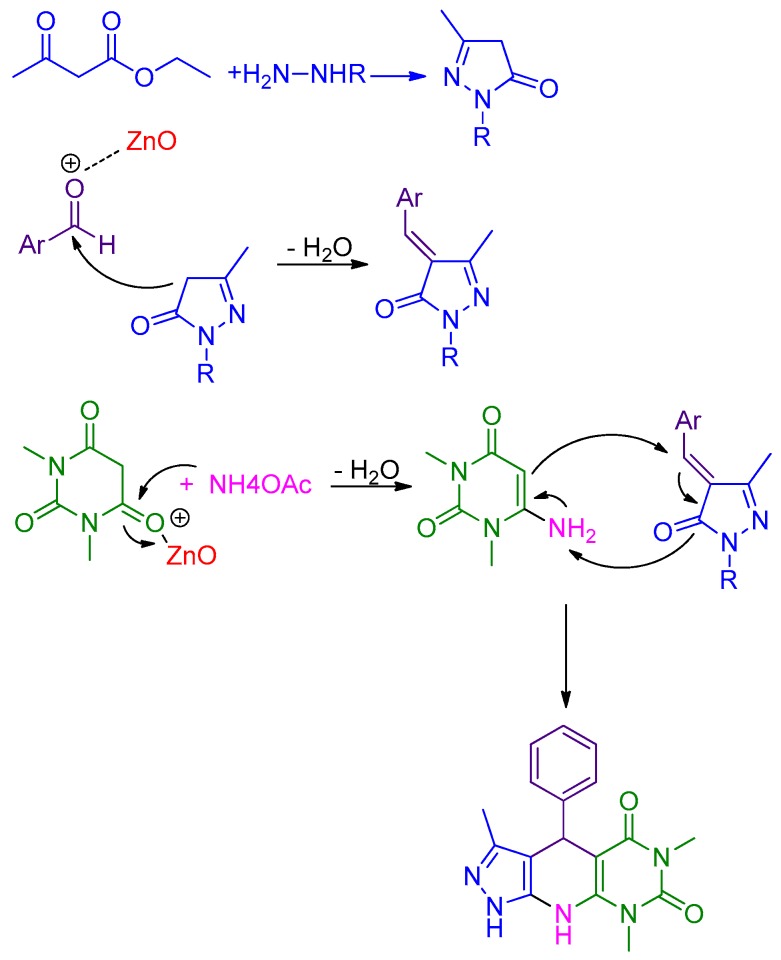
Proposed mechanism for nano ZnO catalyst.

The proposed mechanism for a five-component reaction is summarized in Scheme 3.

For experimental purposes, initially, hydrazine hydrate (1.1 mmol) was added drop wise to ethyl acetoacetate (1 mmol) and then an already prepared mixture of nano ZnO, benzaldehyde, 1,3-dimethyl barbituric acid and ammonium acetate was added. This mixture was refluxed in water. The progress of our model reaction was monitored by TLC. Upon completion of the reaction, the corresponding pyrazolo-[4′,3′:5,6]pyrido[2,3-*d*]pyrimidine-dione was obtained in high yield. (Table 3).

The product was deposited in the water to form a separate filtrate, thus consuming much energy to evaporate the solvent being non-required. Upon filtering of the cold reaction mixture, the Zno nano-catalyst remains on the top of funnel along with product. Upon recrystallization of the product from CH_2_Cl_2_, it can be recovered, recycled and reused with a simple washing up, at least in three consecutive runs without appreciable loss of activity.

As is clear from the structure, there is a chiral center in the final product. Since our suggested mechanism involves sequential Knoevenagel–Michael addition reactions that can be catalyzed in both acidic and basic conditions, we thought it was worthwhile to examine asymmetric synthesis of pyrazolo-[4′,3′:5,6]pyrido[2,3-*d*]pyrimidine-diones via asymmetric organocatalysis.

l-proline is a readily obtainable naturally occurring amino acid. It has also been reported as an eco-friendly catalyst for the synthesis of several heterocycles [64,65,66]. We thought it was worthwhile to examine it as an asymmetric basic organocatalyst to attain optical activity in our products.

**Scheme 4:**
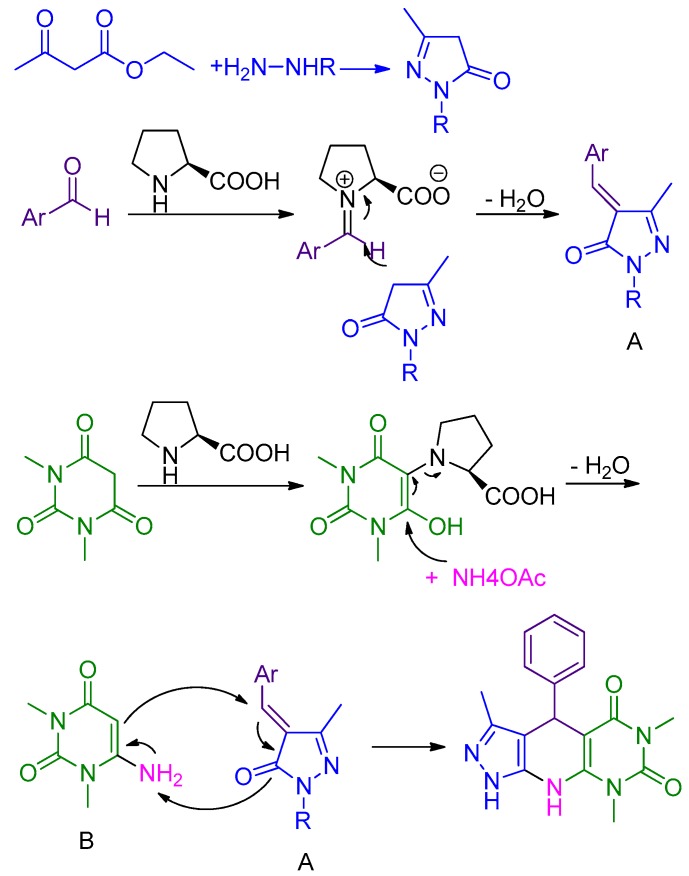
Proposed mechanism for the synthesis of compound **6** using l-proline.

Since several asymmetric synthesis using l-proline has been done at room temperature [67], initially, the model reaction was performed at 25 °C to afford compound **6a**. Unfortunately, the yield of the product was very low, so the reaction mixture was heated at reflux temperature for the indicated times. Although the reaction proceeded smoothly leading to the desired product, we did not observe any optical activity in the isolated product, perhaps due to reflux conditions. To establish the generality of proline-catalyzed reaction, various aromatic aldehydes were used to give the desired products it worked as an ordinary base (Scheme 4). However, the results obtained from the nano-ZnO-catalyzed reaction had overshadowed this approach.

The corresponding product was produced in high yield, but unfortunately does not show any optical activity. The results are summarized in Table 4.

## 3. Experimental

Melting points were measured by using the capillary tube method with an electro thermal 9200 apparatus. ^1^H-NMR and ^13^C-NMR spectra were recorded on a Bruker spectrometer (Ettlingen, Germany) at 400 MHz, respectively, using TMS (Tetramethylsilane) as an internal standard (DMSO solution). IR spectra were recorded from KBr disk on the FT-IR spectrometer (Ettlingen, Germany) Bruker Tensor 27. The reactions were monitored by TLC. All solvents and reagents were purchased from Aldrich (Taufkirchen, Germany) and Merck (Darmstat, Germany) with high-grade quality, and used without any purification. All products are new and were fully characterized by their spectral and physical data (Please find Supplementary Materials for more details).

### 3.1. General Procedure

#### 3.1.1. Synthesis of Pyrazolo-[4′,3′:5,6]pyrido[2,3-*d*]pyrimidine-diones: Using 4-Component Reaction

To a mixture of 3-methyl-pyrazol-5-one or 3-methyl-1-phenyl-pyrazole-5-one (1 mmol), 1,3-dimethyl barbitueic acid (1 mmol), ammonium acetat (1.2 mmol) and benzaldehyde (1 mmol), a catalytic amount of nano-ZnO (0.04 g) was added and the resulting mixture was heated at reflux in H_2_O (5 mL). The progress of the reaction was monitored by TLC. On completion, the mixture was cooled and filtered. The precipitate was recrystallized from CH_2_Cl_2_ to give pure target compounds. All the products were identified by comparison of their physical and spectroscopic data with those reported for authentic samples. The physical and spectral data are given.

#### 3.1.2. Synthesis of Pyrazolo-[4′,3′:5,6]pyrido[2,3-*d*]pyrimidine-diones Using 5-Component Reaction

Initially, hydrazine hydrate (1.1 mmol) was added drop wise to ethyl acetoacetate (1 mmol) and then other components containing 1,3-dimethyl barbitueic acid (1 mmol), ammonium acetat (1.2 mmol), benzaldehyde (1 mmol), ZnO nanoparticles (0.04 g) were added at once, and the mixture was heated at reflux temperature for the time indicated in Table 3. After completion of the reaction (as monitored by TLC), the mixture was cooled and filtered. The precipitate was recrystallized from CH_2_Cl_2_ to give pure target compounds.

#### 3.1.3. Synthesis of Pyrazolo-[4′,3′:5,6]pyrido[2,3-*d*]pyrimidine-diones: Using 4-Component Reaction

A solution of 3-methyl-pyrazol-5-one or 3-methyl-1-phenyl-pyrazole-5-one (1 mmol), 1,3-dimethyl barbitueic acid (1 mmol), ammonium acetat (1.2 mmol), benzaldehyde (1 mmol) and l-proline (0.04 g) in H_2_O (5 mL) was stirred under heating conditions for appropriate time. After completion of the reaction which was monitored by TLC, the mixture was cooled to room temperature. The solid product was collected by filtration, washed with water and aqueous ethanol and purified by recrystallization from CH_2_Cl_2_.

### 3.2. Spectral Data

*3,6,8-Trimethyl-4-phenyl-8,9-dihydro-1H-pyrazolo[4′,3′:5,6]pyrido[2,3-d]pyrimidine-5,7(4H,6H)-dione (Entry 1, Table 2)*. m.p.: 194–196 °C; IR (KBr, ν_max_, cm^−1^): 3332, 2971, 1654, 1248. ^1^H-NMR (400 MHz, DMSO-*d*_6_): δ 2.22 (s, 3H, CH_3_), 2.81 (s, 3H, CH_3_), 3.07 (s, 3H, CH_3_), 5.63 (s, 1H, CH), 7.006–7.084 (m, 3H, Ar-H), 7.158–7.196 (t, 2H, *J* = 7.2, Ar-H), 10.46 (brs, 2H, 2-NH).

*4-(4-Fluorophenyl)-3,6,8-trimethyl-8,9-dihydro-1H-pyrazolo[4′,3′:5,6]pyrido[2,3-d]pyrimidine-5,7(4H,6H)-dione (Entry 2, Table 2).* m.p.: 281–283 °C; IR (KBr, ν_max_, cm^−1^): 3312, 2956, 1663, 1280. ^1^H-NMR (400 MHz, DMSO-*d*_6_): δ 1.96 (s, 3H, CH_3_), 3.06 (s, 3H, CH_3_), 3.46 (s, 3H, CH_3_), 4.99 (s, 1H, CH), 7.14–7.16 (d, *J* = 7.6 Hz, 2H, Ar-H), 7.36–7.38 (d, *J* = 7.6 Hz, 2H, Ar-H), 9.81 (s, 1H, NH), 11.99 (s, 1H, NH); ^13^C-NMR (100 MHz, DMSO-*d*_6_) δ: 10.02, 27.15, 30.28, 33.84, 88.63, 106.23, 125.06, 130.41, 134.57, 135.63, 137.31, 142.28, 149.62, 150.19, 158.24; calcd for C_17_H_16_FN_5_O_2_: C, 57.07; H, 4.51; N, 19.57; found: C, 57.12; H, 4.50; N, 20.02

*4-(4-Chlorophenyl)-3,6,8-trimethyl-8,9-dihydro-1H-pyrazolo[4′,3′:5,6]pyrido[2,3-d]pyrimidine-5,7(4H,6H)-dione (Entry 3, Table 2)*. m.p.: 285–285 °C; IR (KBr, ν_max_, cm^−1^): 3321, 2948, 1647, 1263.

*4-(4-Bromophenyl)-3,6,8-trimethyl-8,9-dihydro-1H-pyrazolo[4′,3′:5,6]pyrido[2,3-d]pyrimidine-5,7(4H,6H)-dione (Entry 4, Table 2)*. m.p.: 286–288 °C; IR (KBr, ν_max_, cm^−1^): 3329, 2948, 1644, 1261.

*4-(2,4-Dichlorophenyl)-3,6,8-trimethyl-8,9-dihydro-1H-pyrazolo[4′,3′:5,6]pyrido[2,3-d]pyrimidine-5,7(4H,6H)-dione (Entry 5, Table 2)*. m.p.: 285–287 °C; IR (KBr, ν_max_, cm^−1^): 3341, 2937, 1640, 1258.

*4-(4-nitrophenyl)-3,6,8-trimethyl-8,9-dihydro-1H-pyrazolo[4′,3′:5,6]pyrido[2,3-d]pyrimidine-5,7(4H,6H)-dione (Entry 6, Table 2)*. m.p.: 172–173 °C; IR (KBr, ν_max_, cm^−1^): 3320, 2949, 1671, 1269.

*3,6,8-Trimethyl-4-(3-nitrophenyl)-8,9-dihydro-1H-pyrazolo[4′,3′:5,6]pyrido[2,3-d]pyrimidine-5,7(4H,6H)-dione (Entry 7, Table 2)*. m.p.: 221–223 °C; IR (KBr, ν_max_, cm^−1^): 3325, 2951, 1643, 1271.

*4-(4-Hydroxyphenyl)-3,6,8-trimethyl-8,9-dihydro-1H-pyrazolo[4′,3′:5,6]pyrido[2,3-d]pyrimidine-5,7(4H,6H)-dione (Entry 8, Table 2)*. m.p.: 220–222 °C; IR (KBr, ν_max_, cm^−1^): 3318, 2963, 1617, 1280.

*4-(4-(Dimethylamino)phenyl)-3,6,8-trimethyl-8,9-dihydro-1H-pyrazolo[4′,3′:5,6]pyrido[2,3-d]pyrimidine-5,7(4H,6H)-dione (Entry 9, Table 2)*. m.p.: 222–224 °C; IR (KBr, ν_max_, cm^−1^): 3331, 2949, 1631, 1271.

*3,6,8-Trimethyl-1,4-diphenyl-8,9-dihydro-1H-pyrazolo[4′,3′:5,6]pyrido[2,3-d]pyrimidine-5,7(4H,6H)-dione (Entry 10, Table 2)*. m.p.: 208–210 °C; IR (KBr, ν_max_, cm^−1^): 3315, 2936, 1650, 1241. ^1^H-NMR (400 MHz, DMSO-*d*_6_): δ 1.55 (s, 3H, CH_3_), 2.70 (s, 3H, CH_3_), 2.82 (s, 3H, CH_3_), 5.09 (s, 1H, CH), 7.42–7.46 (t, *J* = 8.0 Hz, 6H, Ar-H), 7.87–7.89 (d, *J* = 8.0 Hz, 4H, Ar-H), 11.77 (s, 1H, NH).

*3,6,8-Trimethyl-4-(4-fluorophenyl)-1-phenyl-8,9-dihydro-1H-pyrazolo[4′,3′:5,6]pyrido[2,3-d]pyrimidine-5,7(4H,6H)-dione (Entry 11, Table 2)*. m.p.: 207–208 °C; IR (KBr, ν_max_, cm^−1^): 3324, 2949, 1647, 1270.

*4-(4-Chlorophenyl)-3,6,8-trimethyl-8,9-dihydro-1H-pyrazolo[4′,3′:5,6]pyrido[2,3-d]pyrimidine-5,7(4H,6H)-dione (Entry 12, Table 2)*. m.p.: 200–202 °C; IR (KBr, ν_max_, cm^−1^): 3321, 2946, 1641, 1273. ^1^H-NMR (400 MHz, DMSO-*d*_6_): δ 1.96 (s, 3H, CH_3_), 2.88 (s, 3H, CH_3_), 3.46 (s, 3H, CH_3_), 4.86 (s, 1H, CH), 7.14–7.19 (m, 5H, Ar-H), 7.54–7.56 (d, 2H, *J* = 7.6 Hz, Ar-H), 7.96–7.98 (d, 2H, *J* = 7.6 Hz, Ar-H), 11.97 (s, 1H, NH); ^13^C-NMR (100 MHz, DMSO-*d*_6_) δ : 10.91, 26.55, 29.13, 32.69, 87.17, 101.45, 117.40, 122.81, 124.54, 126.33, 127.91, 128.17, 129.21, 134.52, 135.71, 145.43, 147.38, 149.09, 161.98; calcd for C_23_H_20_ClN_5_O_2_: C, 66.18; H, 4.83; N, 16.78; found: C, 66.34; H, 4.76; N, 16.18.

*4-(4-Bromophenyl)-3,6,8-trimethyl-1-phenyl-8,9-dihydro-1H-pyrazolo[4′,3′:5,6]pyrido[2,3-d]pyrimidine-5,7(4H,6H)-dione (Entry 13, Table 2)*. m.p.: 160–161 °C; IR (KBr, ν_max_, cm^−1^): 3317, 2944, 1641, 1279.

*3,6,8-Trimethyl-4-(4-nitrophenyl)-1-phenyl-8,9-dihydro-1H-pyrazolo[4′,3′:5,6]pyrido[2,3-d]pyrimidine-5,7(4H,6H)-dione (Entry 14, Table 2)*. m.p.: 155–157 °C; IR (KBr, ν_max_, cm^−1^): 3330, 2954, 1648, 1266.

*3,6,8-Trimethyl-1-phenyl-4-(p-tolyl)-8,9-dihydro-1H-pyrazolo[4′,3′:5,6]pyrido[2,3-d]pyrimidine-5,7(4H,6H)-dione (Entry 15, Table 2)*. m.p.: 205–206 °C; IR (KBr, ν_max_, cm^−1^): 3315, 2938, 1640, 1259.

## 4. Conclusions

In summary, herein, we report a high yielding one-pot synthesis of pyrazolo-[4′,3′:5,6]pyrido[2,3-*d*]pyrimidine-dione derivatives from the condensation of ethyl acetoacetate, hydrazine hydrate or phenyl hydrazine, 1,3-dimethyl barbituric acid, aryl aldehydes and ammonium acetate catalyzed by nano ZnO and l-proline under triply green conditions, including using green catalysis in water via MCR reaction. The conditions are mild and a wide range of functional groups can be tolerated. Using nano-ZnO as catalyst offers advantages including simplicity of operation, easy work-up and high yields of products. This work will not only lead to establishing a practical synthetic method but will also expand the versatility of clean organic reactions in water.

## Figures and Tables

**Table 1:** Optimization of the reaction conditions for the synthesis of pyrazole based pyrido[2,3-*d*]pyrimidine-diones under thermal conditions.

Entry	Catalyst	Solvent	Time (h)	Yield (%)
1	H_6_P_2_W_18_O_62_·18H_2_O	Water	2	60
2	Sulfamic acid	Water	2.5	65
3	DABCO	Water	3.5	85
4	ZnO	Water	5	80
4	Nano-ZnO	Water	0.66	94
5	Nano-ZnO	EtOH	1	80

**Table 2:** One pot, four-component synthesis of pyrazolo-[4′,3′:5,6]pyrido[2,3-*d*]pyrimidine-diones in refluxing water using a catalytic amount of nano-ZnO.

Entry	Ar	R	Time (h)	Yield (%) ^a^	M.p. (°C)	M.p. rep. (°C) [26]
**1**	C_6_H_5_	H	3	94	194–196	195–196
**2**	4-FC_6_H_4_	H	2.5	90	281–283	------
**3**	4-ClC_6_H_4_	H	2.5	94	285–286	284–285
**4**	4-BrC_6_H_4_	H	2.8	92	286–288	286–287
**5**	2,4-ClC_6_H_3_	H	3.2	92	285–287	286–287
**6**	4-NO_2_C_6_H_4_	H	3	94	172–173	173–174
**7**	3-NO_2_C_6_H_4_	H	3.2	91	221–223	220–221
**8**	4-OHC_6_H_4_	H	4	87	220–222	219–220
**9**	4-N(Me)_2_C_6_H_4_	H	3.8	89	222–224	221–223
**10**	C_6_H_5_	Ph	3.5	92	208–210	210–211
**11**	4-FC_6_H_4_	Ph	3	89	207–208	208–210
**12**	4-ClC_6_H_4_	Ph	3	94	200–202	------
**13**	4-BrC_6_H_4_	Ph	3	92	160–161	158–159
**14**	4-NO_2_C_6_H_4_	Ph	3.7	88	155–157	156–157
**15**	4-CH_3_C_6_H_4_	Ph	4	90	205–506	207–209

^a^ isolated yield.

**Table 3:** One pot, five-component synthesis of pyrazolo-[4′,3′:5,6]pyrido[2,3-*d*]pyrimidine-diones in refluxing water using a catalytic amount of nano-ZnO.

Entry	Ar	R	Time (h)	Yield (%) ^a^	M.p. (°C)	M.p. rep. (°C) [26]
**1**	C_6_H_5_	H	4	91	194–196	195–196
**2**	4-FC_6_H_4_	H	3.5	90	281–283	------
**3**	4-ClC_6_H_4_	H	3.5	91	285–286	284–285
**4**	4-BrC_6_H_4_	H	3.8	90	286–288	286–287
**5**	2,4-ClC_6_H_3_	H	4	90	285–287	286–287
**6**	4-NO_2_C_6_H_4_	H	4	91	172–173	173–174
**7**	3-NO_2_C_6_H_4_	H	4.2	89	221–223	220–221
**8**	4-OH-C_6_H_4_	H	4.5	85	220–222	219–220
**9**	4-N(Me)_2_C_6_H_4_	H	4.5	85	222–224	221–223

^a^ isolated yield.

**Table 4:** One pot, four-component synthesis of pyrazolo-[4′,3′:5,6]pyrido[2,3-*d*]pyrimidine-diones in refluxing water using a catalytic amount of nano-ZnO.

Comp.	Ar	R	Time (h)	Yield (%) ^a^	M.p. (°C)	M.p. rep. (°C) [26]
**1**	C_6_H_5_	H	4	91	195–197	195–196
**2**	4-FC_6_H_4_	H	3.5	90	280–281	------
**3**	4-ClC_6_H_4_	H	3.5	91	283–285	284–285
**4**	4-BrC_6_H_4_	H	4	90	284–286	286–287
**5**	2,4-ClC_6_H_3_	H	4.5	89	286–288	286–287
**6**	4-NO_2_C_6_H_4_	H	4.2	90	171–173	173–174
**7**	3-NO_2_C_6_H_4_	H	4.5	88	220–223	220–221
**8**	4-OHC_6_H_4_	H	5	84	218–221	219–220
**9**	4-N(Me)_2_C_6_H_4_	H	4.2	86	223–224	221–223
**10**	C_6_H_5_	Ph	6	89	209–212	210–211
**11**	4-FC_6_H_4_	Ph	5.5	87	207–209	208–210
**12**	4-ClC_6_H_4_	Ph	5.5	90	201–204	------
**13**	4-BrC_6_H_4_	Ph	6	90	157–159	158–159
**14**	4-NO_2_C_6_H_4_	Ph	5.2	84	154–157	156–157
**15**	4-CH_3_C_6_H_4_	Ph	5.5	85	206–508	207–209

^a^ isolated yield.

## Supplementary Materials

Supplementary materials can be accessed at: http://www.mdpi.com/1420-3049/21/4/441/s1.
