# Supplementary material for: A Novel and Efficient Five-Component Synthesis of Pyrazole Based Pyrido[2,3-d]pyrimidine-diones in Water: A Triply Green Synthesis
Source: Molecules. 2016 Apr 1;21(4):441. doi: 10.3390/molecules21040441 (PMC6274230; doi:10.3390/molecules21040441)
Supplement: Supplementary file 1 [file molecules-21-00441-s001.pdf]

Supplementary Materials: A Novel and Efficient Five-Component Synthesis of Pyrazole Based Pyrido[2,3-*d*]pyrimidine-diones in Water: A Triply Green Synthesis

Majid M. Heravi and Mansoureh Daraie

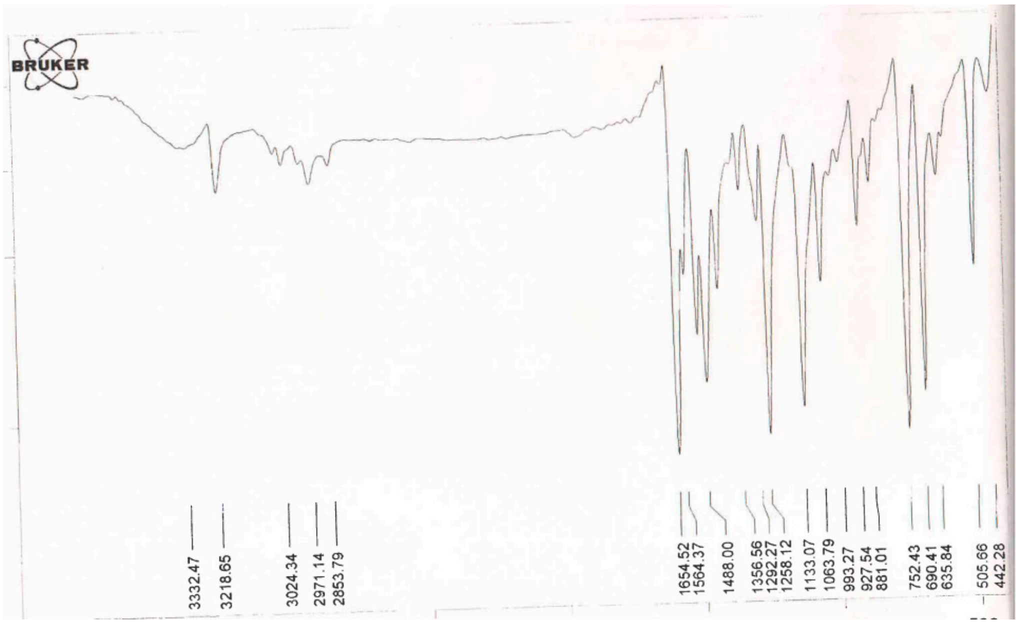

Figure S1. The FTIR spectrum of compund (Entry 1-Table 2).

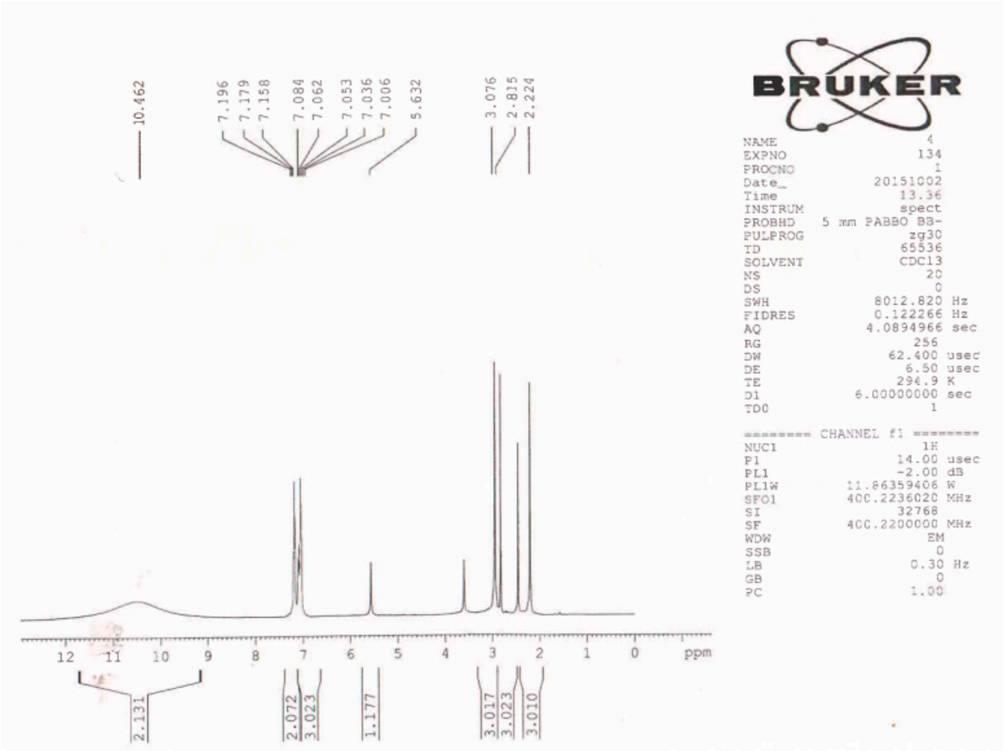

Figure S2. The ^{1}H-NMR spectrum of compund Entry 1-Table 2).

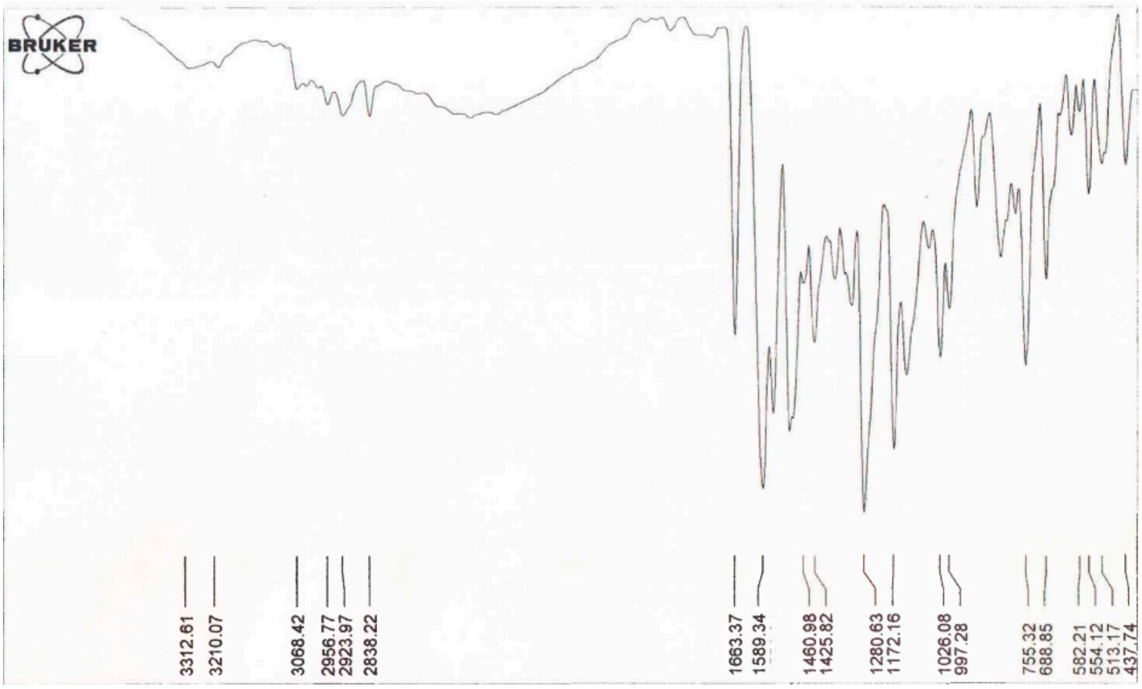

Figure S3. The FTIR spectrum of compund (Entry 2-Table 2).

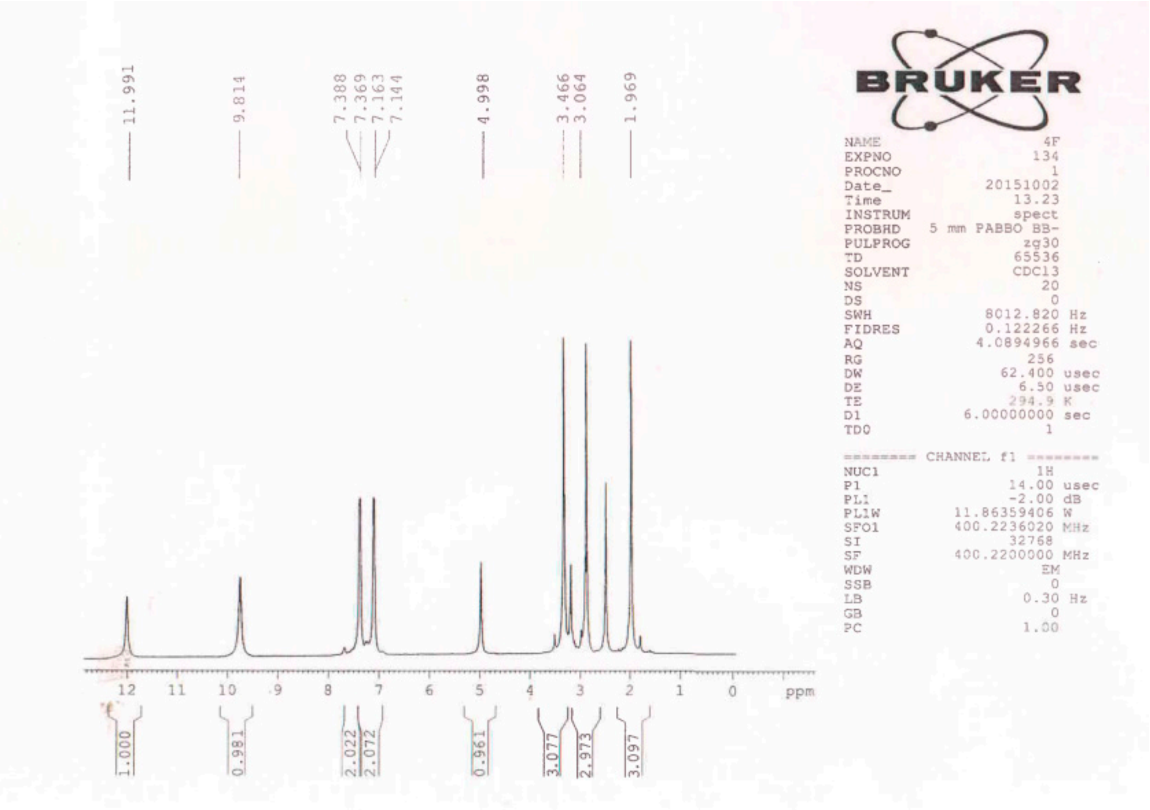

Figure S4. The ^{1}H-NMR spectrum of compund (Entry 3-Table 2).

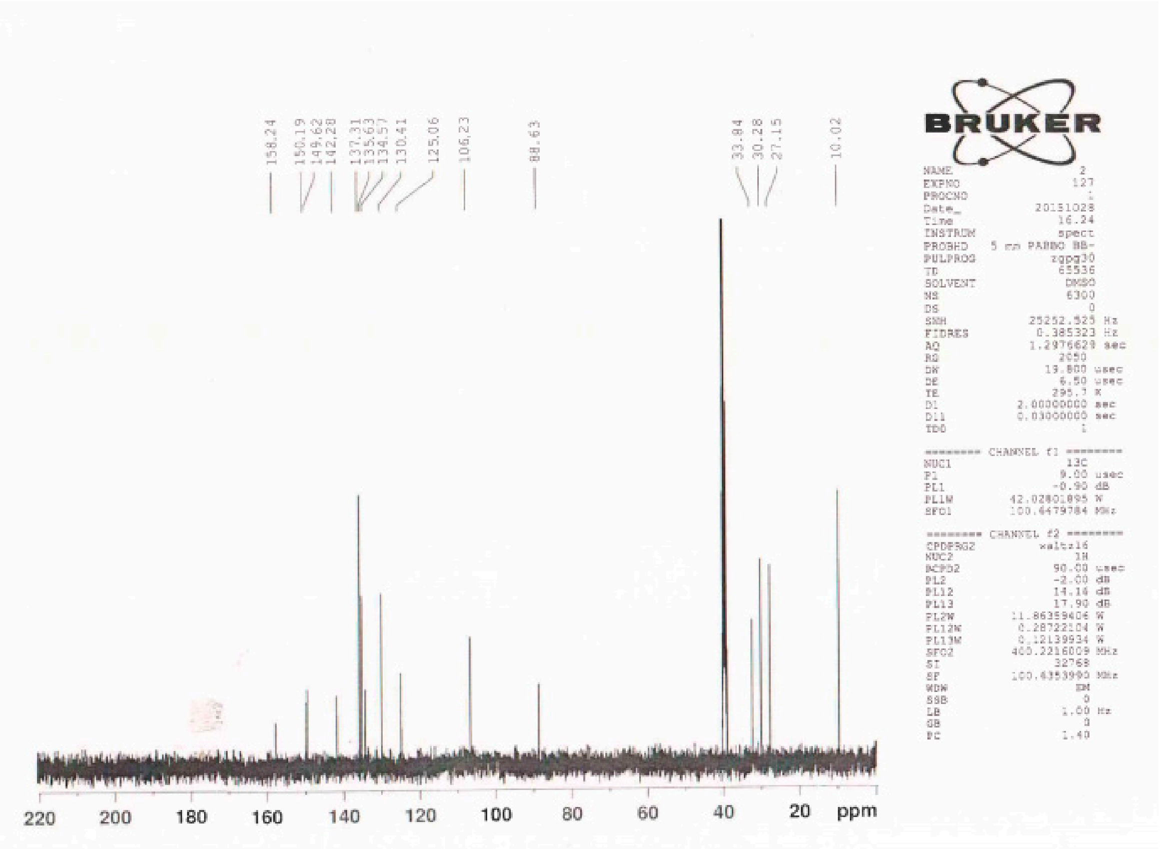

Figure S5. The [13] C-NMR spectrum of compund Entry 3-Table 2)

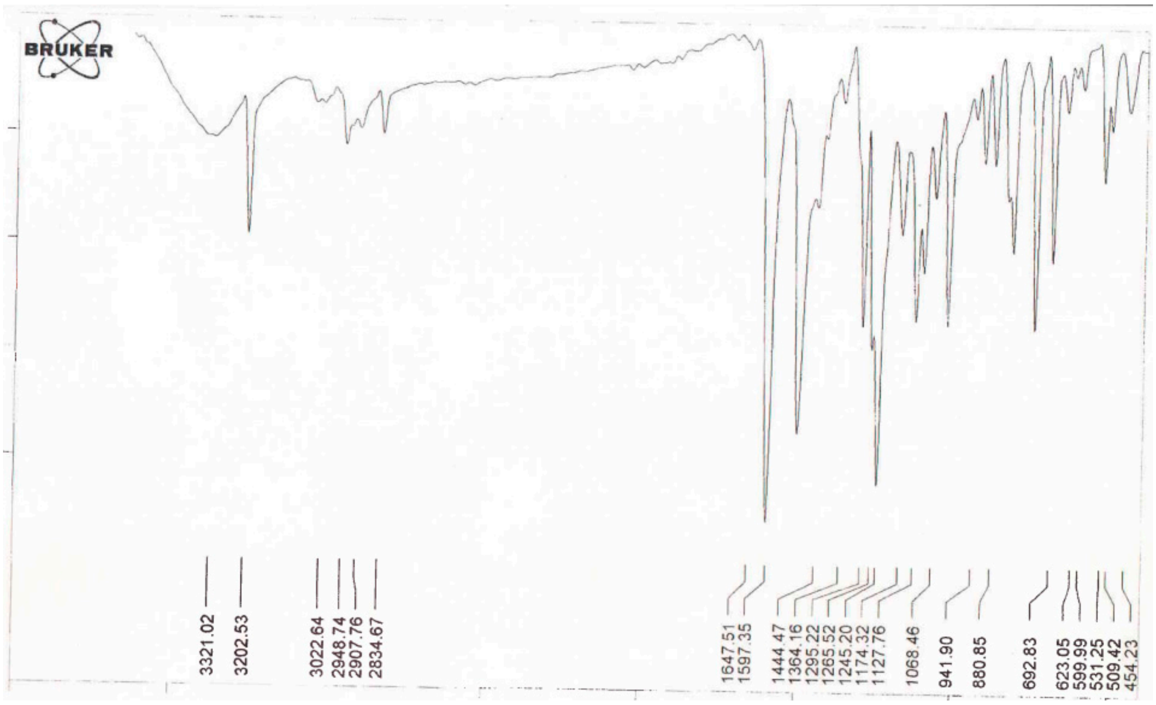

Figure S6. The FTIR spectrum of compund (Entry 4-Table 2).

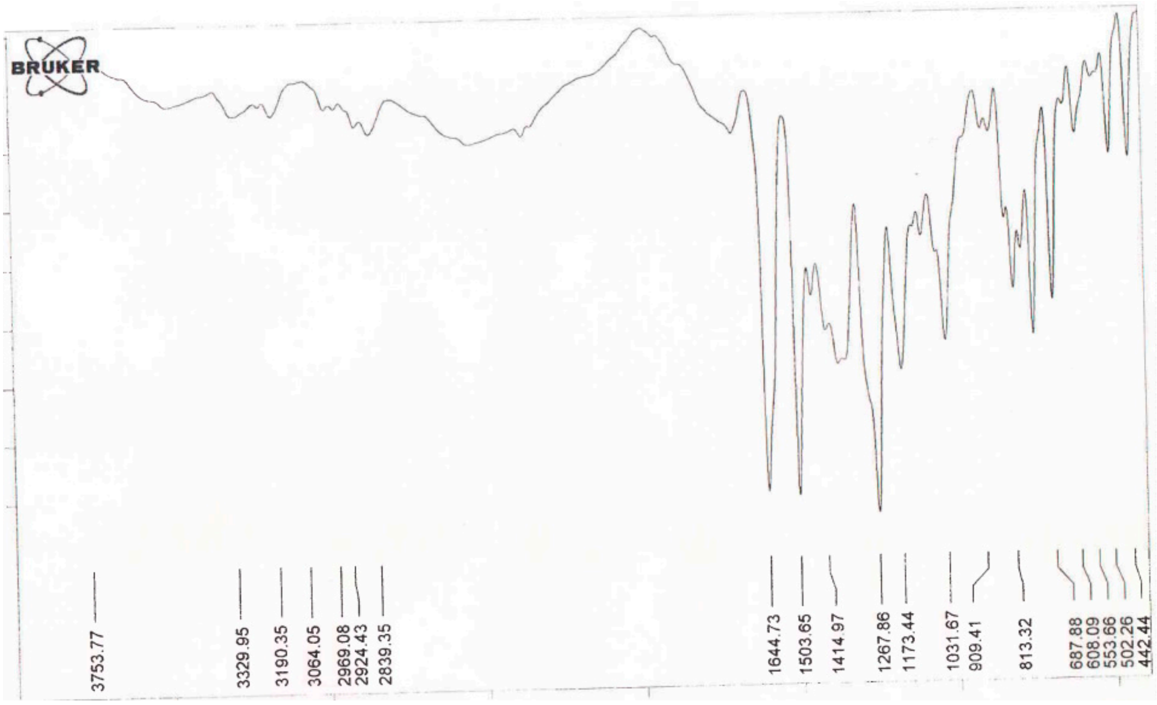

Figure S7. The FTIR spectrum of compund (Entry 5-Table 2).

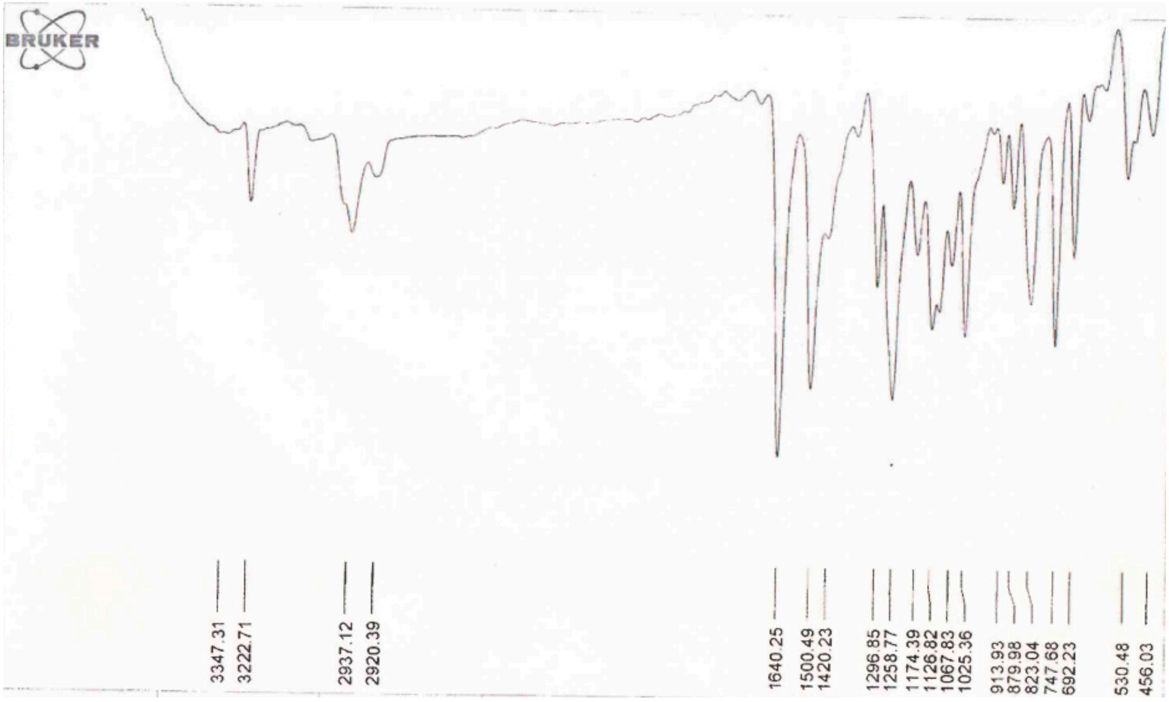

Figure S8. The FTIR spectrum of compund (Entry 6-Table 2).

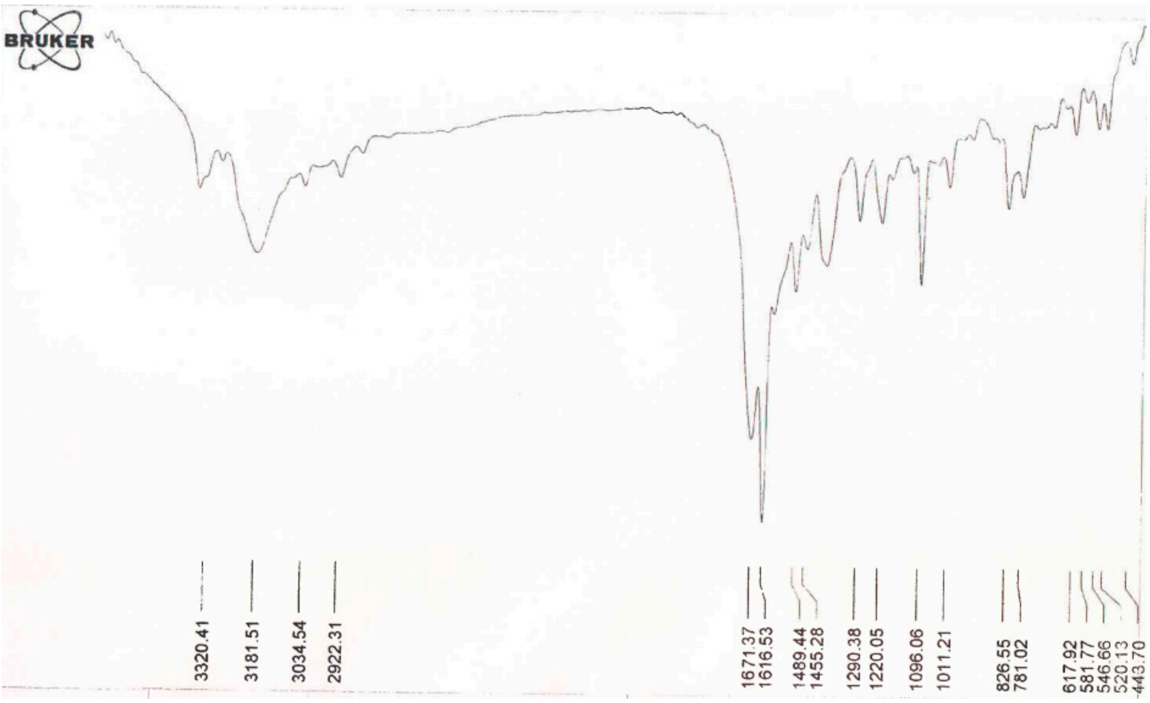

Figure S9. The FTIR spectrum of compund (Entry 6-Table 2).

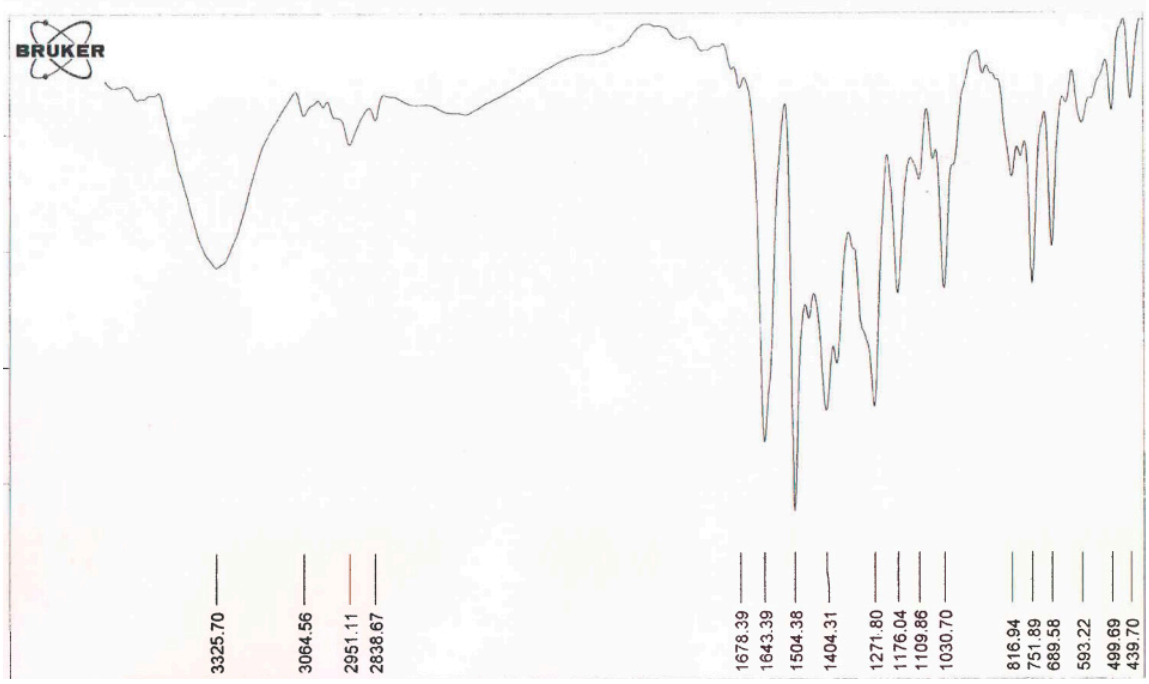

Figure S10. The FTIR spectrum of compund (Entry 7-Table 2).

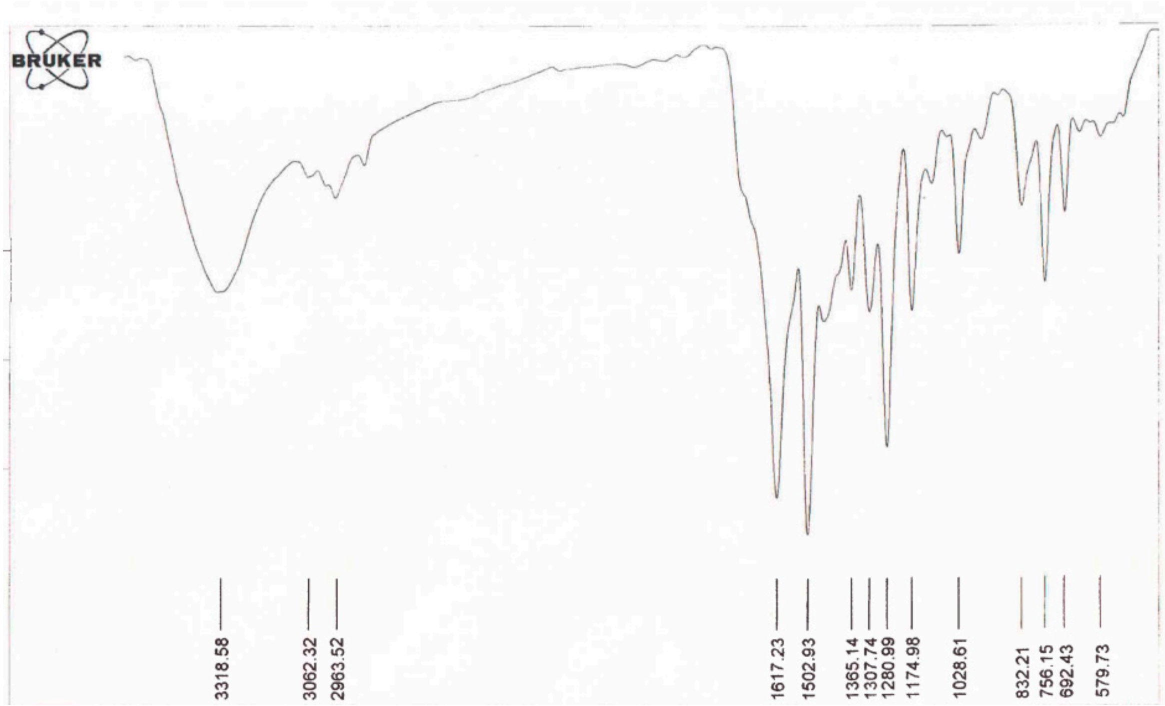

Figure S11. The FTIR spectrum of compund (Entry 8-Table 2).

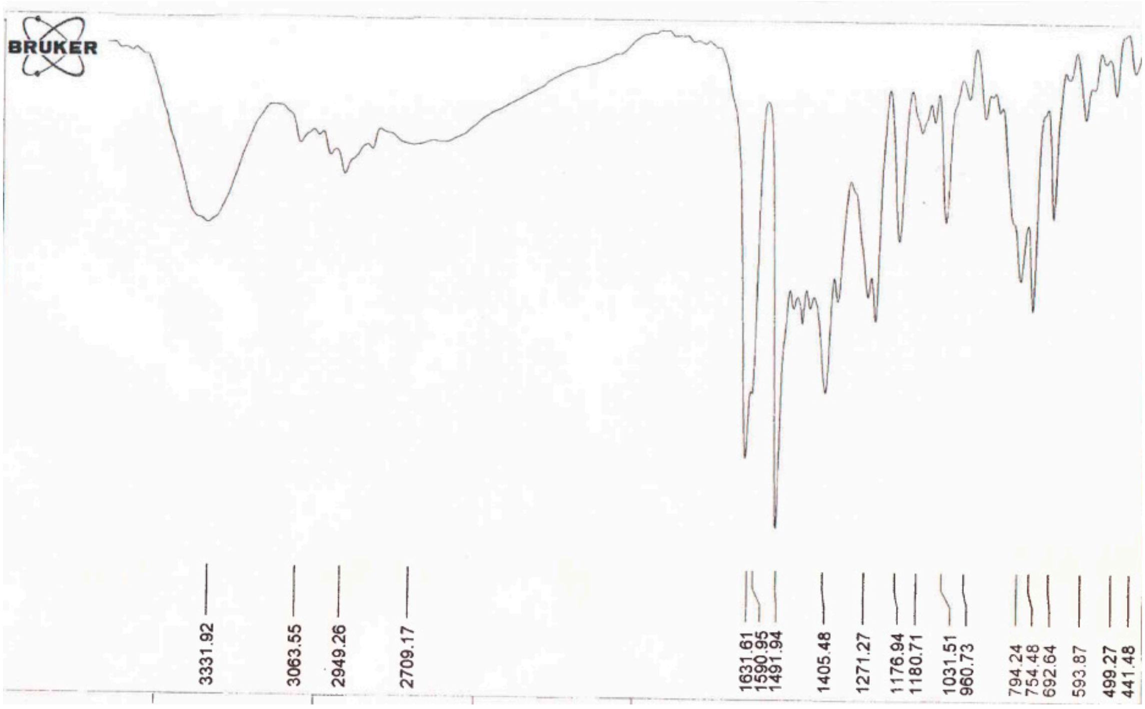

Figure S12. The FTIR spectrum of compund (Entry 9-Table 2).

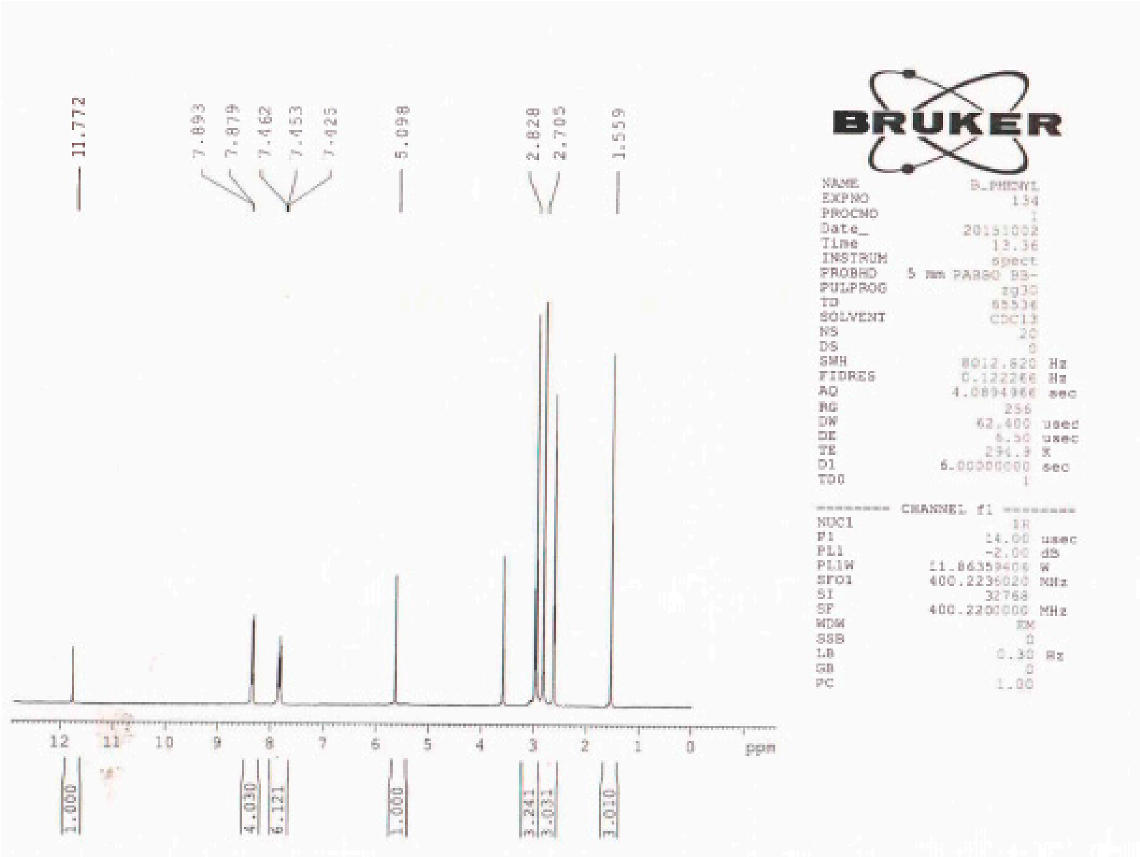

Figure S13. The ^{1}H-NMR spectrum of compund (Entry 10-Table 2).

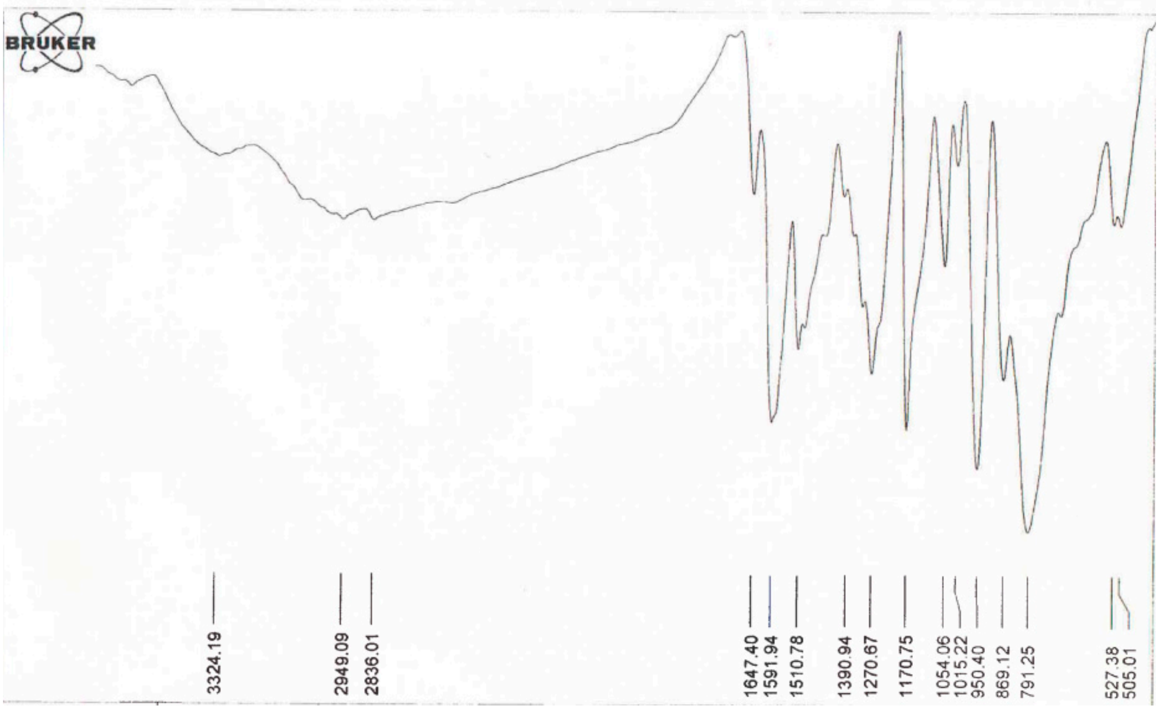

Figure S14. The FTIR spectrum of compund (Entry 11-Table 2).

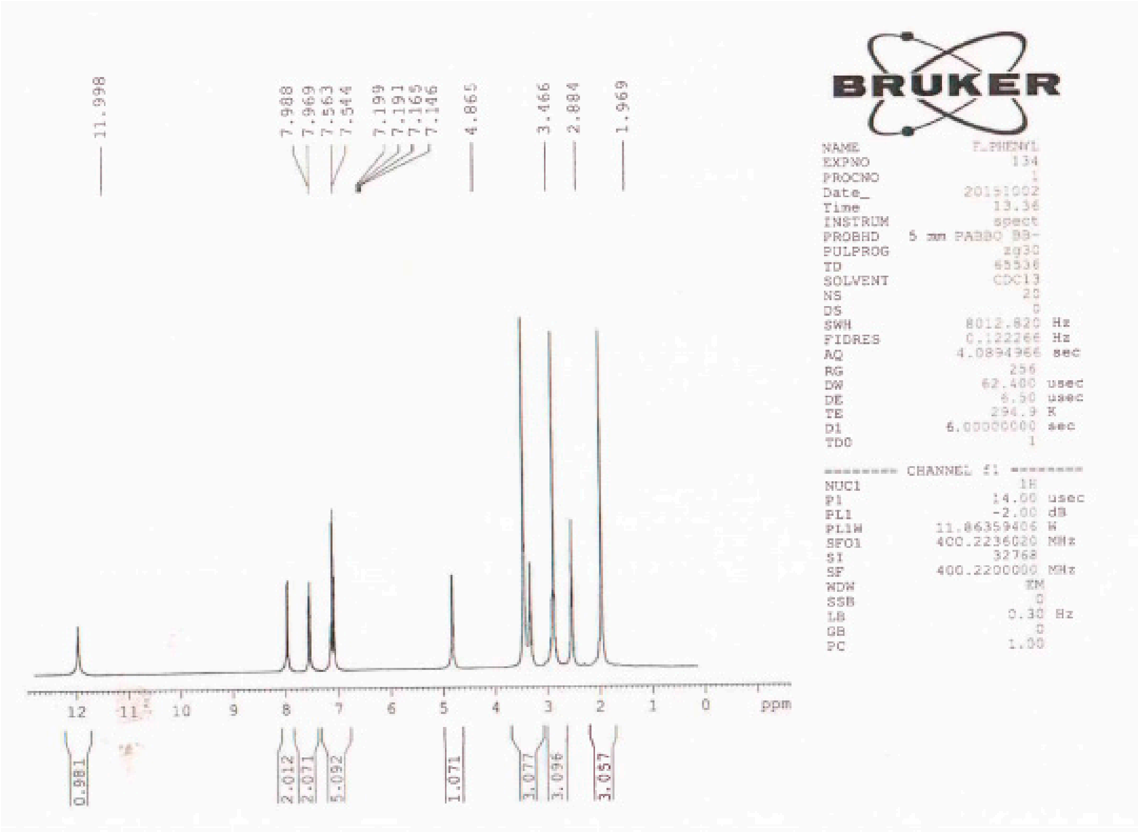

Figure S15. The ^{1}H-NMR spectrum of compund (Entry 12-Table 2).

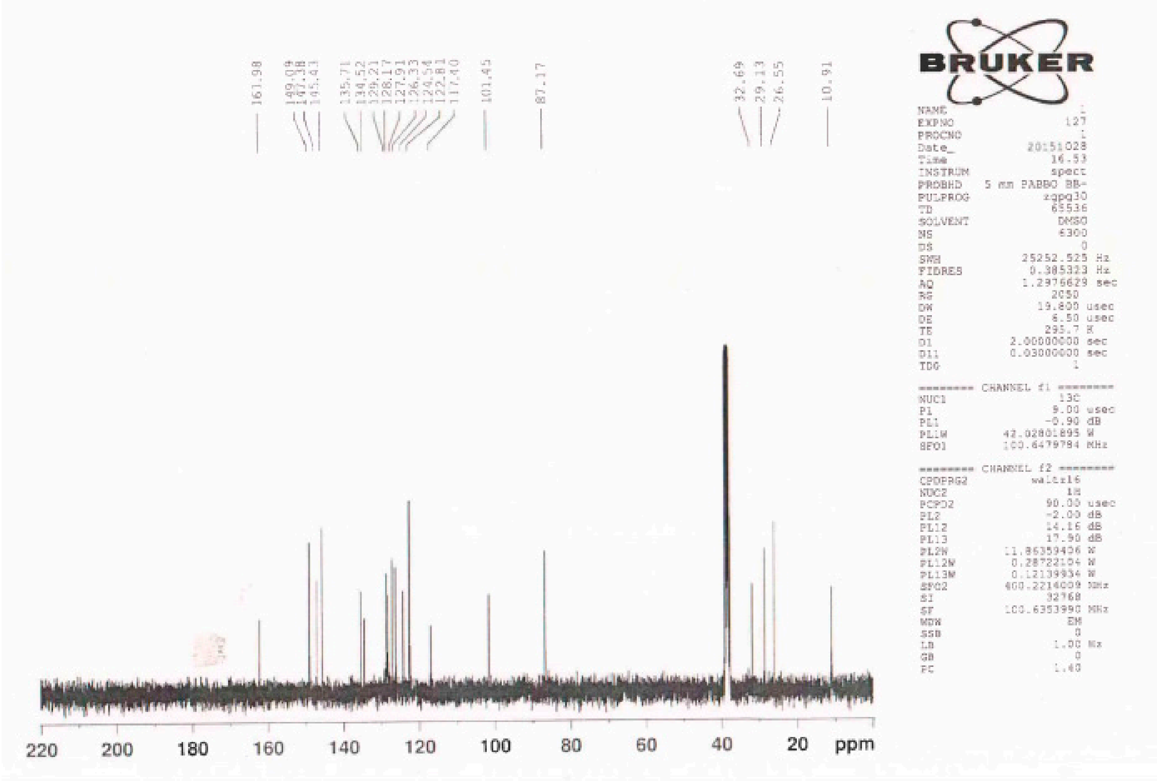

Figure S16. The [13] C-NMR spectrum of compund (Entry 12-Table 2).

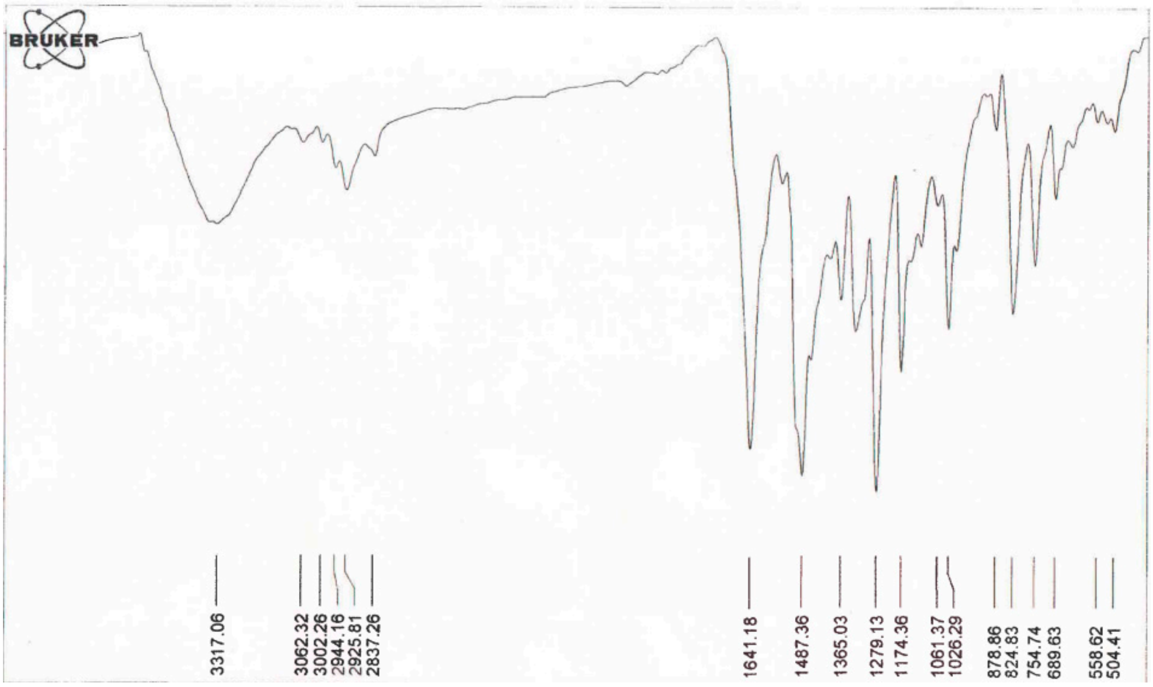

Figure S17. The FTIR spectrum of compund (Entry 13-Table 2).

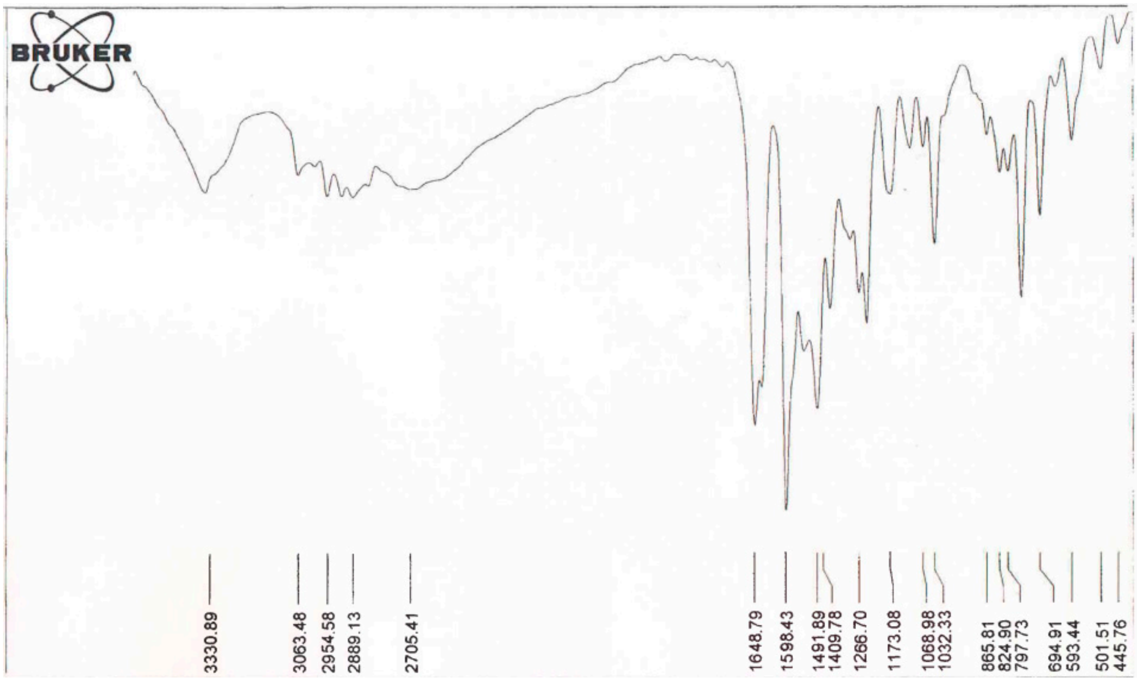

Figure S18. The FTIR spectrum of compund (Entry 14-Table 2).
